# Long-Term Safety of Rapid Daratumumab Infusions in Multiple Myeloma Patients

**DOI:** 10.3389/fonc.2020.570187

**Published:** 2020-12-21

**Authors:** Alessandro Gozzetti, Francesca Bacchiarri, Vincenzo Sammartano, Marzia Defina, Anna Sicuranza, Bianca Mecacci, Elisabetta Zappone, Emanuele Cencini, Alberto Fabbri, Donatella Raspadori, Monica Bocchia

**Affiliations:** Hematology Unit, University of Siena, Azienda Ospedaliero Universitaria Senese, Siena, Italy

**Keywords:** Daratumumab, multiple myeloma, rapid infusion, safety, efficacy

## Abstract

Multiple myeloma survival has significantly improved in recent years, due to novel agents that are available for treatment. The anti-CD38 monoclonal antibody Daratumumab is particularly efficient for patients with relapse/refractory disease, and many studies have shown its unprecedented efficacy also as a first treatment. However, to avoid the incidence of infusion reactions, long infusion schedules of 8 h at first dose and 4 h in the following doses are required, which can reduce the compliance of patients and health care professionals. A reduced infusion time of 90 min has been reported previously, but data are missing on the prolonged safety of this over time as well as the efficacy of this approach. In this work, we investigate the safety of 484 rapid Daratumumab infusions given early after the second dose over a 22 months period in 39 myeloma patients.

## Introduction

Multiple myeloma (MM) is a plasma cell disorder, the prognosis of which has dramatically improved in the last years thanks to new immunomodulatory drugs (IMIDs), proteasome inhibitors (PIs), and monoclonal antibodies (mAbs), in relapsed/refractory disease and at diagnosis (1–6). Daratumumab, a CD38 antagonist, is a human immunoglobulin G1 kappa (IgG1κ) monoclonal antibody that binds CD38 expressed on the cell surface in a variety of hematological malignancies, including myeloma, lymphomas, and leukemias, as well as on other cell types and tissues with various expression levels. Daratumumab acts through different mechanisms of action including an immune-mediated effect: i) antibody-dependent cytotoxicity (ADC), ii) complement-dependent cytotoxicity (CDC), and iii) antibody-dependent cellular phagocytosis (ADCP). It can also cause apoptosis through a direct antitumor effect or it can act as an immune modulator to decrease immunosuppression or infuence CD38 enzymatic inhibition. When Daratumumab was used as a single drug in relapsed/refractory MM patients who previously received a median of four lines of therapy, it led to an unprecedented response of 31% (ORR) and a median OS of 20.1 months (7). CASTOR and POLLUX studies demonstrated that the addition of Daratumumab to bortezomib and lenalidomide, respectively, prolonged PFS and OS in the relapsed refractory setting (8, 9). Daratumumab combinations with pomalidomide and carfilzomib have been used for relapsed refractory disease, and other combinations are now approved also at diagnosis (10–14). In particular, the combination with pomalidomide and dexamethasone has been tested in a cohort of heavily pretreated patients, giving an ORR of 60% (17% CR or better), while Daratumumab combined with carfilzomib and dexamethasone did not reach PFS at a median follow-up of 17 months. In the Alcyone trial (12) Daratumumab–VMP (velcade, melphalan, prednisone) compared to VMP alone showed a superior PFS (36.4 vs 19.3 months, respectively) and OS (78% vs 68.9%, respectively, at 40 months of follow up) in newly diagnosed MM patients (NDMM) that were not eligible for transplantation. Furthermore, the MAIA study (13) showed the superiority of Daratumumab in NDMM patients when comparing Dara–Rd (Daratumumab, Revlimid, Dexamethasone) with Rd alone. PFS was superior for Dara–Rd vs Rd (not reached vs 31.9 months), with 24% of minimal residual disease (MRD) showing negativity in the Dara–Rd group. Finally, the Cassiopeia study (14) evaluated Daratumumab–VTD (velcade, thalidomide, Dexamethasone) vs VTD in NDMM patients in pre-transplant induction therapy and post-transplant consolidation. Dara–VTD was superior in terms of the depth of response (MRD negativity of 64% vs 44%) and PFS (93% vs 85% at 18 months). These studies showed the good tolerability of daratumumab; however, due to the expression of CD38 on airway smooth muscle cells, infusion-related reactions (IRRs) are very common at the first dose, accounting for about 40% of patients and presenting with symptoms (cough, wheezing, rhinorrhea) similar to those of allergic rhinitis. Daratumumab as a monotherapy or when combined with revlimid dexamethasone is given intravenously (IV), every week for 8 weeks, then every 2 weeks for 16 weeks, and then every month, while the Dara–Velcade regimen (DVd) is given weekly with 9 doses, then every 3 weeks with 5 doses, and then every 4 weeks (15). It is worthy of note that the infusion duration is particularly long for the first infusion (7 h), with median durations of 4.3 and 3.5 h for second and other infusions, respectively. Moreover, if an infusion-related reaction presents, the infusion may take longer, worsening the patient’s quality of life and also affecting the planning of daily practice for physicians and nurses. Splitting the first dose over two days reduces the length of each infusion (4 h each), although the patient has to come to the hospital twice. Strategies to reduce the infusion time have been reported (16): in one study, the authors showed safety in 28 patients with MM with an infusion time of 90 min starting from a dose >10 in the majority of cases. In 11 out of 28 patients, a 90 min infusion was given from the third dose. No report, however, has been made regarding the prolonged safety of rapid Daratumumab infusions over time, nor regarding the efficacy of the drug with this modified schedule. It is worthy of note that the subcutaneous formulation has been tested in many trials and is about to be introduced in clinical practice (17).

## Materials and Methods

### Patient Characteristics

From December 2018 to October 2020, we performed an observational prospective single-arm, single-center study, with the twofold aim of reducing the time of infusion and the stay of the patient in the day hospital. The primary endpoint of the study was the safety of rapid Daratumumab infusions. The secondary endpoint was the safety of the first Daratumumab infusions. Patients receiving Daratumumab as standard clinical practice at relapse and patients enrolled in clinical trials at diagnosis could be enrolled. Patient characteristics are reported in **Table 1**. Nineteen patients were affected by RRMM and received Daratumumab alone or in combination with other agents (lenalidomide, bortezomib, pomalidomide), and 20 patients received Daratumumab as consolidation therapy in the protocol DART4MM (this trial was registered at ClinicalTrials.gov with the number NCT039922170; EUDARACT N. 2018-000386-36: Daratumumab as consolidation therapy in patients who already achieved >VGPR/MRD positivity during first line therapy). Ethical approval was obtained locally, and DART4MM was also approved by the Italian Dug Regulatory Agency (AIFA, with a specific approval amendment for rapid Daratumumab infusions).

**Table 1:** Patient characteristics.

N	M/F	Age	Type	FISH:S/H	N.Previous lines	Dara-therapy	N.Dara previous infusions	IRR1 dose	N.Rapid infusions	IRR in Rapid infusions	Disease status before Dara	Bestresponse	PD:Y/N	FUO/infection	A/D
1	M	68	IgA κ	S	3	DVd	16	No	**4**	**None**	RR	PR	Y	Yes	Dead
2	M	84	IgG κ	S	6	DRd	3	No	**28**	**None**	RR	PR	N	Yes	Alive
3	M	57	IgG κ	ND	4	DVd	5	No	**18**	**None**	RR	PR	N	Yes	Alive
4	M	60	BJ.κ	H	6	DRd	3	No	**24**	**None**	RR	PR	Y	Yes	Dead
5	M	53	IgG κ	H	2	DPd	2	Yes	**12**	**None**	RR	SD	Y	Yes	Dead
6	M	71	IgG κ	N	1	DRd	2	No	**21**	**None**	RR	CR	N	No	Alive
7	M	58	BJ.λ	S	1	DRd	2	Yes	**19**	**None**	RR	PR	N	Yes	Alive
8	F	62	IgG λ	N	6	Dara alone	13	No	**1**	**None**	RR	PR	Y	Yes	Dead
9	M	57	IgG λ	N	3	DRd	5	Yes	**7**	**None**	RR	SD	Y	No	Dead
10	M	65	IgG λ	N	1	DRd	2	Yes	**4**	**None**	RR	VGPR	N	Yes	Alive
11	M	63	BJ λ	N	1	Dara alone	2	No	**19**	**None**	RR	VGPR	N	Yes	Alive
12	M	63	IgG λ	ND	1	Dara alone	12	No	**18**	**None**	RR	PR	N	No	Alive
13	M	49	IgG/k	H	6	Dara Alone	13	No	**4**	**None**	RR	PR	Y	No	Dead
14	F	55	IgG/k	H	2	DVd	2	No	**11**	**None**	RR	PR	N	No	Alive
15	F	63	IgG/k	S	6	DRd	2	No	**9**	**None**	RR	VGPR	N	No	Alive
16	M	64	BJ/λ	S	2	DRd	2	No	**7**	**None**	RR	VGPR	N	No	Alive
17	M	45	IgG/k	S	1	DRd	2	No	**1**	**None**	RR	NA	N	No	Alive
18	M	61	IgG/k	S	4	DVd	2	No	**1**	**None**	RR	NA	N	No	Alive
19	F	63	BJ/λ	S	1	DRd	2	Yes	**1**	**None**	RR	NA	N	No	Alive
20	F	51	IgG κ	S	1	**DART4MM**	2	No	**21**	**None**	CR-MRD pos	CR	N	No	Alive
21	F	63	IgG κ	S	1	**DART4MM**	2	No	**14**	**None**	CR-MRD pos	CR	N	No	Alive
22	M	71	IgA λ	S	1	**DART4MM**	2	No	**14**	**None**	VGPR	sCR-MRD neg	N	No	Alive
23	M	62	IgG k	S	1	**DART4MM**	2	No	**16**	**None**	CR-MRD pos	CR	N	No	Alive
24	F	65	IgG k	S	1	**DART4MM**	2	No	**16**	**None**	VGPR	VGPR	N	No	Alive
25	F	72	IgG λ	H	1	**DART4MM**	3	Yes	**26**	**None**	VGPR	VGPR	Y	No	Alive
26	F	78	IgG κ	N	1	**DART4MM**	2	Yes	**30**	**None**	VGPR	CR	N	No	Alive
27	M	71	IgG κ	S	1	**DART4MM**	2	No	**14**	**None**	VGPR	sCR-MRD neg	N	No	Alive
28	M	66	IgG λ	ND	1	**DART4MM**	2	No	**14**	**None**	VGPR	CR-MRD neg	N	No	Alive
29	F	65	IgG λ	S	1	**DART4MM**	2	Yes	**14**	**None**	CR-MRD pos	CR-MRD neg	N	No	Alive
30	M	55	BJ λ	ND	1	**DART4MM**	2	No	**14**	**None**	CR-MRD pos	CR-MRD neg	N	No	Alive
31	M	58	IgG κ	S	1	**DART4MM**	2	Yes	**14**	**None**	VGPR	CR-MRD neg	N	No	Alive
32	M	48	IgG λ	S	1	**DART4MM**	2	No	**14**	**None**	CR-MRD pos	CR-MRD neg	N	No	Alive
33	M	70	IgG/k	S	1	**DART4MM**	2	No	**18**	**None**	VGPR	VGPR	N	No	Alive
34	F	66	IgG/k	S	1	**DART4MM**	2	No	**12**	**None**	VGPR	CR	N	No	Alive
35	M	65	IgG/k	S	1	**DART4MM**	2	Yes	**15**	**None**	VGPR	VGPR	N	No	Alive
36	M	66	IgG k	S	1	**DART4MM**	2	No	**5**	**None**	VGPR	NA	N	No	Alive
37	M	62	IgG/k	S	1	**DART4MM**	2	Yes	**2**	**None**	CR-MRD pos	NA	N	No	Alive
38	M	52	IgG/k	S	1	**DART4MM**	2	Yes	**1**	**None**	VGPR	NA	N	No	Alive
39	F	59	IgG/k	S	1	**DART4MM**	2	Yes	**1**	**None**	VGPR	NA	N	No	Alive

FISH : ND, not done; H = high risk (t 4;14-t14;16-del 17p); S, standard risk (all other anomalies); N, normal; DVd, dara, velcade, dexamethasone; DRd, Dara, Revlimid, dexamethasone; DPd, Dara, Pomalidomide, dexamethasone; NA, not applicable (too early for assessment); IRR, infusion-related reaction; FUO, fever of unknown origin.

N Rapid infusions and IRRR in Rapid infusions columns are evidenced in bold.

### Treatment Schedule

The infusion schedule is reported in **Table 2**. The first dose of daratumumab was split into day 1 and day 2 with a median infusion duration of 4 h each. Rapid infusion could start from the third dose if no IRR was seen in the previous infusion (second). A rapid infusion rate was calculated to deliver 20% of the dose over 30 min (200 ml/h), and then the rate was increased to deliver the remaining 80% over 60 min (450 ml/h). This resulted in a 90 min estimated infusion time (total volume 550 ml). Premedication drugs, according to the current clinical practice, were corticosteroids (IV dexamethasone 20 mg), antipyretic (oral paracetamol 1.000 mg), and antihistamine (IV chlorphenamine maleate 10 mg); a two-puff inhalation of β-adrenergic bronchodilator (salbutamol) given 20 min before the first infusion was chosen instead of montelukast after a referral with a local pneumologist. All patients received antiviral and antibacterial prophylaxis (Acyclovir 400 mg twice daily, trimethoprim–sulfamethoxazole, 160 + 800 mg twice daily twice/week). All patients treated with lenalidomide or pomalidomide received thromboprophylaxis with low-dose aspirin (18). Safety assessments included any adverse events (AEs), physical examinations, clinical laboratory parameters, vital signs measurement, and Eastern Cooperative Oncology Group performance status. AEs were assessed using the National Cancer Institute Common Terminology Criteria for Adverse Events Version 4.03 (19). Responses were defined according to IMWG criteria (20). Minimal residual disease (MRD) was evaluated by next-generation flow on bone marrow aspirate with a sensitivity of 10^-6^ (Euroflow criteria) (21).

**Table 2:** Infusions schedule.

Daratumumab	Total volume	Initial infusion rate	Increments of infusion rate	Maximum infusion rate	Total infusion time
I infusion:					
Day 1 + Day 2	500 ml + 500 ml	50 ml/h	50 ml/h every hour	200 ml/h	4 h
II infusion	500 ml	50 ml/h	50 ml/h every hour	200 ml/h	4 h
>III infusion	550 ml	200 ml/h for 30 min	450 ml/h for 60 min	450 ml/h	1,5 h

## Results

A total of 39 consecutive patients (27 M/12 F) with a median age of 64 years (range 48–84 years) were referred to our Hematology center and received a rapid infusion of daratumumab, leading to a total of 484 rapid infusions (median number 14, range 1–30) over a 22 month observational period (**Table 1**). No serious adverse event was recorded in all patients during rapid infusion. Six out of 39 (15%) patients (one with associated renal amyloidosis) had been previously treated with Daratumumab on entry to the study. They had already received a median of 12 infusions (range 5–16), and no IRRs were registered during subsequent rapid infusions (**Table 3**). Pre-existing chronic obstructive pulmonary disease or asthma were absent in previous patient history in 38 out of 39 patients. One patient reported smoking activity in their past history; they were then referred to the pneumologist, and a therapy with β−adrenergic bronchodilator was administered three days before the first infusion.

**Table 3:** Summary of main patient characteristics and results.

**Patients N**	**39**
M/F	27/12
Age median (range)	64 (48–84)
Study period	December 2018–October 2020
Disease status:	
Relapse	19
Dara Schedule:	
DRd	10
DVd	4
Dara alone	4
DPd	1
Consolidation (DART4MM)	20
IRR at first infusion	13/39 (33%)
Grade 1–2	13 (100%)
Allergic rhinitis/nasal congestion	7 (54%)
Dyspnoea	3 (13%)
Chills	3 (13%)
Total N. Rapid infusions	484
Median N. rapid infusions per patient (range)	14 (1-30)
N. Patients starting Dara Rapid since Third infusion	30/39
IRR in rapid infusions	0/484

### Infusion-Related Reactions at First Dose During Daratumumab Long-Infusion Treatment

IRRs at the first Daratumumab split infusion were seen in 13 of 39 patients (33%): five were in RR MM patients, while the other 8 patients were included in the DART4MM study. All IRRs at the first dose happened on day 1; they were mild and of grades 1–2: (seven showed allergic rhinitis, three showed dyspnea, and three exhibited chills), and the drug was temporarily stopped in all cases. Allergic rhinitis was treated with IV hydrocortisone hemisuccinate at 100/200 mg, chlorphenamine maleate, and salbutamol. All bronchospasms were treated with salbutamol aerosol: in one case, IV chlorphenamine maleate at 10 mg was also added, and in two cases, hydrocortisone hemisuccinate at 200 mg was also added. Chills were treated with corticosteroids IV in all cases. The median time to recovery after IRRs was 30 min. All patients who had an IRR during the first split dose did not have other reactions during subsequent infusions, regardless of the infusion time.

### Infusion-Related Reactions at Rapid Infusions

Thirty-three of 39 (79%) patients received Daratumumab as a rapid infusion upfront (30 at the third infusion, day 15, cycle 1; and three patients at the fourth infusion, day 22, cycle 1). No subsequent IRRs were seen. Premedication was reduced after dose >10, when Daratumumab was used as a monotherapy or in the DART4MM study, with oral antihistamine (loratadine 10 mg), dexamethasone 10 mg (IV), and paracetamol 500 mg (oral) in all patients. Moreover, in 6 patients, corticosteroids were reduced to 4 mg dexamethasone after dose >14.

### Adverse Events That Were Not Infusion Related

Treatment-emergent adverse events (TEAEs) during therapy and that were not infusion-related were similar to other populations of patients treated with daratumumab, all of grades 1–2 (Hematological TEAEs: lymphopenia in 12 patients, 31%; anemia in 9, 23%; thrombocytopenia in 9, 23%; neutropenia in 9, 23%. Non-hematological TEAEs: arthralgia in 8 patients, 20%; fever of unknown origin (FUO) in 8 patients, 20%). Two patients who received Daratumumab in combination with lenalidomide had pneumonia/infection and stopped treatment: one patient had a septic shock requiring emergency care unit admission—at last follow-up, they are alive and in VGPR—the other patient had bacterial pneumonia (staphylococcus aureus), maintaining a stable response. Both patients had grade 3 neutropenia at the time of hospitalization.

### Response to Treatment

During Daratumumab therapy, 23 of 32 evaluable patients had a response improvement (72%). In particular, in the DART4MM protocol, 9 of 16 patients showed an improved response, from a VGPR or CR-MRD pos to a CR or CR-MRD neg by next-generation flow, while in the evaluable RRMM patients, 14 of 16 had at least a PR. Six of 39 patients exhibited a halted progression: three patients were treated at line 6, two at line 3 and one patient at line 2 of therapy, respectively. The latter maintained an SD for 6 months with Daratumumab before progressing (who received Daratumumab for 6 months while maintaining a SD).

## Discussion

In this study, we describe the largest population of MM patients so far reported to receive rapid infusions of Daratumumab from cycle 1 day 15 (n = 30) with the longest follow-up to evaluate safety. In fact, the long follow-up period reported of 22 months makes the study quite reliable to evaluate both short and long-term safety. The availability of Daratumumab has revolutionized treatment practice for MM, leading to a marked improvement in outcomes for the treatment of MM patients. However, the current administration schedules can negatively impact patient’s compliance and out-patient care system organization. The rapid infusion Daratumumab schedule here reported provides a practical and safe solution that has a positive impact on resource utilization. Another point of interest is that rapid infusion was revealed to be particularly convenient during the Covid-19 emergency, reducing patients’ time spent in hospitals and reducing social contacts for patients and staff. Rapid infusions have been tested with other monoclonal antibodies in the past; for example, rapid 60 min infusions of rituximab (IgG1 chimeric anti CD20 antibody) for B cell lymphoproliferative disorders have been adopted at our institution and many others since 2006 (22, 23). The efficacy of rituximab was maintained and patients were rapidly dismissed from the clinic. Rapid Daratumumab infusion has very recently been reported in another trial (24) in which 53 MM patients received rapid infusions and no difference was seen in terms of IRRs with respect to 47 patients receiving long infusions (1.9% vs 4.3%). The latter study compared rapid infusions vs standard IV infusions: 53 patients received rapid infusions of Daratumumab, but only 18 received the rapid infusion starting from cycle 1 day 15 upfront (as in our study). Importantly, the authors showed a cost saving for rapid infusions: the total costs estimated for a 52 week regimen of Daratumumab infused at standard and rapid rates were $137,200 and $122,200 (P <.001), respectively. However, in comparison to our study, there is no report on the safety of the method in long-term follow-up, nor regarding the efficacy of the drug. Recent studies have also shown the good safety and efficacy of the subcutaneous (SC) infusion with a dose of 1,800 mg with recombinant human hyaluronidase PH20 (rHuPH20)(concentration 30,000 U; in 15 ml) in a single, premixed vial that was administered over 3 to 5 min, and this formulation will be available soon (12, 25). The serum pharmacokinetic (PK) concentrations and efficacy achieved with an 1800 mg SC dose were consistent with what has been observed for Daratumumab 16 mg/kg IV in patients with RRMM. Certainly, SC administration will be more utilized in the near future, but we have to consider that, despite the reduced number of IRRs during SC therapy compared to the IV formulation, almost all reactions happened during the first 6 h after injection. Although PK was not performed in the present study, efficacy was maintained in our study. To our knowledge, no study has so far reported on the efficacy of rapid infusions; however, even considering the small number of patients treated, the treatment efficacy appeared to be quite in line with that expected after the standard infusion length. In conclusion, our study demonstrates that a rapid infusion of Daratumumab after the second dose is safe and well tolerated. As such, while waiting for the SC formulation, this schedule can be adopted by many hematological centers to improve MM patients’ quality of life and allow more sustainable out-patient care infusion planning.

## Data Availability Statement

The raw data supporting the conclusions of this article will be made available by the authors, without undue reservation.

## Ethics Statement

The studies involving human participants were reviewed and approved by Comitato Etico Regionale-Regione toscana, area vasta sud est; Agenzia Italiana del Farmaco. The patients/participants provided their written informed consent to participate in this study.

## Author Contributions

AG designed the study and wrote the manuscript. MD, EZ, AF, and EC were involved in the patient treatment. AS and DR performed the laboratory analysis. FB, VS, and BM collected the data and helped to write the manuscript. MB supervised the study and revised the manuscript. All authors contributed to the article and approved the submitted version.

## Conflict of Interest

The authors declare that the research was conducted in the absence of any commercial or financial relationships that could be construed as a potential conflict of interest.

The handling Editor declared a past co-authorship with one of the authors AG.
